# Active learning methodology, associated to formative assessment, improved cardiac physiology knowledge and decreased pre-test stress and anxiety

**DOI:** 10.3389/fphys.2023.1261199

**Published:** 2023-09-06

**Authors:** Lais Tono Cardozo, Patricia Oliveira de Lima, Maeline Santos Morais Carvalho, Karina Reche Casale, Ana Luisa Bettioli, Maria Antonia Ramos de Azevedo, Fernanda Klein Marcondes

**Affiliations:** ^1^ Department of Biosciences, Piracicaba Dental School, University of Campinas (UNICAMP), Piracicaba, Brazil; ^2^ Department of Education, Institute of Biosciences, Study and Research Group in University Pedagogy, State University of São Paulo (UNESP), Rio Claro, Brazil

**Keywords:** stress, active learning, educational game, group activity, assessment

## Abstract

Stress and anxiety caused by assessments are often related to the student’s insecurity regarding the knowledge to be evaluated, while teaching strategies that increase effective learning can assist in reducing it. The aim of this study was to evaluate the hypothesis that the use of an active methodology, associated to formative assessment, could reduce students’ anxiety and stress, when compared to the traditional method, by promoting greater learning. New students enrolled in the same discipline of a Dentistry course were invited to participate in the study and were divided into two groups: traditional method and active methodology. The traditional method group received two lectures, delivered orally. The active methodology group received a lecture about cardiac cells and the autonomic control of cardiac function, with home study of the cardiac cycle using a textbook. In the second class, an individual formative assessment was applied. Afterwards, a group activity was performed with an educational game about the cardiac cycle, followed by a group formative assessment. After applying the traditional or active methodology, test 1 was carried out. Immediately before this test, saliva samples were collected for determination of the concentrations of the stress biomarkers cortisol and α-amylase. The students also answered the State-Trait Anxiety Inventory questionnaire, used for anxiety level determination. The score obtained in the test 1 was significantly higher for the active methodology group, compared to the traditional method group. No significant differences between the groups were observed for baseline cortisol and salivary α-amylase concentrations, or for anxiety scores. Before test 1, traditional method group presented higher concentrations of salivary cortisol and α-amylase, compared to the respective baseline values, while the active methodology group showed no difference between the baseline and test 1 levels. Before test 1, there were increases in anxiety levels, relative to the respective baseline values, regardless of the teaching methodology used, but this increase was greater for the traditional method group, compared to the active methodology group. These results showed that the active methodology, associated to formative assessment, decreased test stress and anxiety, with improved student performance in comparison to traditional lectures.

## 1 Introduction

For undergraduate students in the area of health, learning about physiology can be a challenge, because it requires the understanding and integration of physiological concepts and knowledge about the morphology of organs and body systems. Considering that this content is extensive and that young people are now accustomed to the use of information and communication technologies (Abdulmajed et al., 2015) and have difficulty in maintaining interest and attention during long theoretical classes (Kulasegaram and Rangachari, 2018; Raes et al., 2019), it is necessary to use other teaching strategies that encourage student engagement. Furthermore, it is essential to create an environment in which the students feel free to make mistakes and learn from colleagues (Martins and Almeida, 2019; Xavier et al., 2020), perceiving the meaning and importance of the topics for their current or future life (Sousa and Alves, 2017). In this way, it is possible to enhance learning and reduce academic stress.

Academic stress, which is the stress related to study, can have negative impacts on student performance (Karaman and Watson, 2017), which may be aggravated when the student feels unable to cope with challenges (Correia et al., 2003). Learning assessments are considered the main situations causing academic stress, due to insecurity regarding the learned content (Pereira et al., 2010). A lack of formative assessments can often mean that students only identify their doubts about the studied content at the time of the test (Kulasegaram and Rangachari, 2018).

The use of active learning methodologies, together with formative assessments, can assist in increasing the learning of students and their confidence in what has been learned. An example of an active learning methodology is the use of educational games, which can contribute to the development of problem-solving, communication, and teamwork skills, while also increasing the interest and motivation of the students (Barclay et al., 2011; Schneider and Jimenez, 2012; Luchi et al., 2017; Marcondes et al., 2022).

For the teaching of cardiac physiology, a cardiac cycle puzzle was developed (Marcondes et al., 2015), which students from different courses in the health area (Dentistry, Medicine, Biology, Physiotherapy, Nursing, and Pharmacy) considered useful for learning, since it assisted in clarifying doubts and facilitated understanding of the relationships between the morphology and physiology of the heart and cardiac functioning (Marcondes et al., 2015). In a study to determine whether this perception of the students was accompanied by learning improvements, Cardozo *et al.* (2016) reported that Dentistry students who had performed the activity with the educational game achieved higher test scores, with fewer incorrect answers, compared to students who had only attended a theoretical lecture. Given these positive effects, another study was performed to evaluate whether the educational game, combined with formative assessments, could increase learning about cardiovascular physiology and reduce the levels of stress and anxiety of students before a test (Cardozo et al., 2020b). The topics considered were the cardiac cycle and blood pressure control, delivered using an active methodology (cardiac cycle puzzle and formative assessments) and theoretical lecture, respectively. The topics had similar levels of difficulty, according to the participating students. Better performance, with lower stress and anxiety, was found for the cardiac cycle test, compared to the blood pressure control test. Although the participants considered that the topics had similar difficulty and the results served to confirm the tested hypothesis, a limitation of the study was that the topics used were not the same.

Therefore, the objective of the present study was to compare the effects of active and traditional methods in learning about the same subject, cardiac physiology, as well as the levels of stress and anxiety caused by assessments, in students of a course in the health area.

## 2 Methods

### 2.1 Ethical considerations and experimental design

**FIGURE 1:**
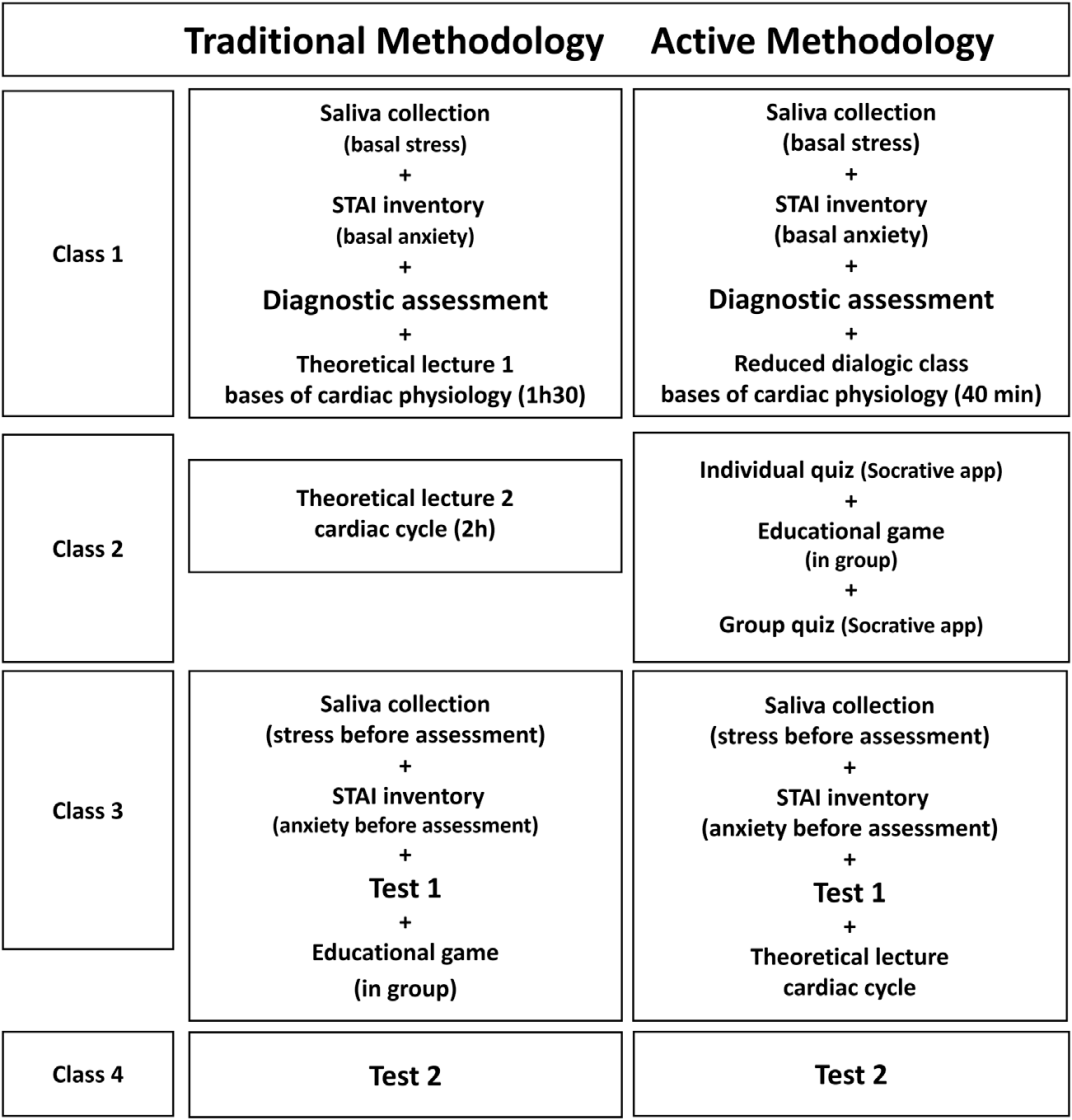
Sequences of teaching strategies and assessments employed in the traditional and active methodologies.

This study was approved by the Research Ethics Committee of Piracicaba Dental School, University of Campinas (CAAE 42980515.0.0000.5418). The participating students were from the first year of the Dentistry course, enrolled in the Biosciences II discipline, in the second semesters of the years 2019 (*n* = 82) and 2022 (*n* = 72). Of these, 73 and 66 students, respectively, agreed to participate in the study and signed an informed consent form. The data considered were for students who participated in all the teaching and assessment activities, did not report any pathology, were not taking the Biosciences II course for the second time or had experience of a human physiology course at another educational institution, and had not completed another higher education course in the area of health. These criteria ensured that the students did not have any prior knowledge of physiology that could facilitate the understanding of the subjects studied, which could have positively influenced their performance in the assessments. Consequently, the data considered were for 52 and 51 students from the years 2019 and 2022, respectively. The teaching of cardiac physiology employed an active methodology in 2019 and a traditional method in 2022, as shown in Figure 1.

The effects of the teaching methodologies on learning were evaluated using the grades obtained by the students in the tests. Stress levels were determined using the concentrations of cortisol and α-amylase in saliva (Lima et al., 2013). Anxiety levels were measured using the State-Trait Anxiety Inventory (STAI) (Biaggio and Natalício, 1979). Baseline stress and anxiety levels were determined in the first physiology class of the semester, in a week when there were no tests, assignments, or other evaluations in any of the disciplines taken by the students. The values obtained were used as controls in determination of the effect of stress on learning. Saliva collection and application of the STAI were performed in 30 min (Figure 1), between 8 and 10 a.m. in order to control the influence of circadian rhythm on cortisol secretion. Diagnostic evaluation was then applied to identify any differences between the classes in terms of the knowledge required for understanding cardiac physiology.

Anxiety and stress measurements were also performed before a cardiac physiology test (test 1, Figure 1). Immediately after this test, each group had a lesson conducted with the other teaching methodology (crossover experimental design). The learning of the students was then evaluated in a second test, after both groups had been exposed to the two different teaching methodologies. If there was a difference between the groups in tests 1 and 2, it could not be explained by the teaching methodology used, since the difference might be related to specific aspects of the groups, such as interest in the topic or dedication to studying.

On the other hand, if there was a difference between the groups in test 1, but not in test 2, the first would probably be related to the teaching method, since the difference was no longer observed when all the students had received both a traditional class and a class using the active methodology. There was no intention of measuring stress and anxiety before test 2, because the aim of this assessment was to control for the effect of any difference between the groups.

### 2.2 Teaching methodologies

The traditional methodology consisted of two theoretical lectures, concerning 1) the fundamentals of cardiac physiology and 2) the cardiac cycle, lasting 1 h 30 min and 2 h, respectively. The first class consisted of an explanation about the characteristics of heart cells, comparison between the action potentials of cardiac and skeletal muscles, control of cardiac function by the autonomic nervous system, and functioning of the heart valves. In the second class, the teacher provided a detailed description of the phases of the cardiac cycle, relating heart muscle contraction to spreading of the electrical stimuli generated in the pacemaker cells, including the roles of conduction fibers, gap junctions, and action potential. The functioning of the heart valves was also described in this class. Afterwards, the students received guidance for home study of the contents addressed in the class, in preparation for a test that would be applied in the next class (Figure 1).

The active methodology employed the sequence of activities and formative assessments shown in Figure 1. The first class was a short dialogic lesson (40 min), where the teacher presented slides and, during the oral explanation, asked questions so that the students could recall topics already studied (action potential, skeletal muscle contraction, and heart structure), relating them to the topics being presented: characteristics of heart cells and valves, comparison between plateau heart muscle action potential (without temporal summation) and skeletal muscle action potential (with temporal summation), and autonomic nervous system control of cardiac function.

Afterwards, the students received guidance for home study of the content addressed in the class, as well as the content of the next class (the cardiac cycle). The teacher explained that after that the first class, the students would be able to understand the events of the cardiac cycle, reading about the topic in the indicated textbook. The students were also informed that individual study was necessary for the group activity that would be performed in the next class, when any doubts remaining after reading the textbook would be resolved.

At the start of class 2, a quiz was applied, using the free application Socrative (https://www.socrative.com), containing questions about the previous class and the cardiac cycle, with a grade given for participation. The use of grades for participation was a strategy to incentivize students to study and prepare for the group activity. Grades were not given for correct and incorrect responses, because it would be in class 2 that the cardiac cycle topic would be studied, with the educational game activity, based on the knowledge acquired in the previous class and by reading the textbook. Therefore, it would not have been appropriate to assess learning by giving grades for correct and incorrect responses, before the teacher had checked and resolved any doubts of the students following their study using the textbook at home. Immediately after the quiz, the class was divided into groups of five to six students for the educational game activity.

It was explained that the purpose of this activity was that the students should apply their knowledge acquired in the individual home study, working together to identify the sequence of events in the cardiac cycle, associating the transmission of the electrical stimulus generated in the pacemaker cells with the characteristics of the conduction fibers, the roles of gap junctions and the plateau action potential, and the participation of the heart valves in the pumping of blood.

The cardiac cycle puzzle consisted of three steps. Firstly, each group of five to six students was asked to identify the correct sequence of 5 figures, provided on A4 paper on a board, illustrating the phases of the cardiac cycle (Marcondes et al., 2015). Next, the students completed a table with the cards provided, indicating the states of the atria and ventricles (contracted or relaxed) and the heart valves (open or closed), as well as the names of the cardiac cycle phases and the moments at which the heart sounds occur (Marcondes et al., 2015; Cardozo et al., 2016).

The students were told to complete the table by consensus, with the group members discussing the topic and convincing their colleagues, so that they could demonstrate their understanding, learn from each other, and develop communication skills. The completion of the table was only checked by the teacher (or the monitors) after all the cards had been positioned. When a card was positioned incorrectly, the group was asked to again discuss the completion of the table, without indicating which card (or cards) had been incorrectly positioned. In this way, the correct completion of the table was achieved as a result of understanding of the topic, rather than by trial and error. During this period, questions were asked by the monitors and the teacher, in order to assist the discussion among the students.

In the third step of the game, once the table had been correctly completed, the groups were asked a series of questions, where each question was only provided after the previous one had been answered, with the aim of ensuring that every student participated in the discussion of each question. The questions used were as follows: 1) How is the electrical stimulus transmitted in the heart during a cardiac cycle? 2) Why is the delay of the electrical stimulus in the atrioventricular node important for cardiac function? 3) How do gap junctions participate in the conduction of the electrical stimulus and in the contraction of the heart muscle? 4) When we listen to the heart, we hear heart sounds that are commonly called “heartbeats”; a) Explain, in detail, how these sounds are generated, and b) evaluate whether it is correct to say that they are “heartbeats.” At the end of the activity, the students (as a group) answered a quiz, and were then told to study for a summative assessment that would be applied in the next class.

At the start of class 3, saliva was collected and the STAI questionnaire was applied, after which the students were submitted to test 1. Immediately after this test, each class received a lesson performed with the other teaching methodology (Figure 1). Hence, the students who had received the theoretical class on the cardiac cycle performed the activity with the educational game and (as a group) answered the quiz at the end of the activity. The students who had already performed the activity with the cardiac cycle game received a theoretical class on the topic. In class 4, test 2 was applied.

### 2.3 Assessment of learning

The diagnostic assessment applied in class 1 consisted of 4 tests about action potential and the contraction of skeletal muscle, which had been studied in the previous semester, and about cardiac anatomy and the trajectory of blood through the heart, which had been addressed in the previous week, in anatomy classes of the same discipline. This assessment was applied for both groups, using the free Socrative application.

The assessment of learning was done by 2 tests in classes 3 and 4 respectively. Test 1 consisted of three essay questions, one objective question, and one question where the students had to identify true and false statements, correcting the false ones. The questions concerned the differences between the excitation-contraction coupling processes of the skeletal and cardiac muscles, events composing the cardiac cycle, heart sounds, and myocardial infarction.

Test 2 considered four situations with altered cardiac function: tachycardia, bradycardia, myocardial infarction, and cardiac fibrillation. The students were required to make associations between the descriptions and the alterations provided, applying their acquired knowledge about cardiac physiology.

Each test had a total of 10 points, with the same tests being applied to the traditional teaching and active methodology groups (Figure 1).

### 2.4 Stress level

For the collection of saliva samples in the classroom, after asepsis of the hands with 70% alcohol, the students received a Salivette^®^ tube containing a sterile cotton swab. They were instructed to place the swab in the oral cavity and move it for 5 min, in order to stimulate salivation. The swab soaked in saliva was then returned to the Salivette^®^ tube, which was placed in a container with ice. Immediately afterwards, the tubes were centrifuged for 2 min, at 1,000 x *g*, and 1 mL aliquots of saliva were stored in Eppendorf tubes, at −70°C, for subsequent measurements of cortisol and α-amylase by immunoenzymatic assay, using commercial Salimetrics kits (Lima et al., 2013). The samples were analyzed in duplicate, according to the manufacturer’s instructions.

### 2.5 Anxiety level

Anxiety level was determined using the State-Trait Anxiety Inventory (STAI) (Biaggio and Natalício, 1979), which was applied in the first class of the semester, before test 1, immediately after collection of the saliva samples.

### 2.6 Students’ perception

At the end of the semester, the students received a form containing the following questions: 1) Was the activity with the educational game useful for your learning? 2) Did performing the individual and group tests in any way change your way of studying and organizing your time? The students were requested to justify their answers.

### 2.7 Statistical analysis

Comparisons between the groups, considering the scores in the diagnostic assessment, test 1, and test 2, were performed using two-factor analysis of variance, considering as factors the methodology (traditional and active) and learning assessment (diagnostic, test 1, test 2), as well as their interaction. The STAI scores and the concentrations of cortisol and α-amylase in saliva, at baseline and before test 1, were compared using two-factor analysis of variance, considering as factors the methodology (traditional and active) and time of evaluation (baseline and test), as well as their interaction. When there was significant interaction, differences were evaluated using Tukey’s test. The significance level considered was 5%.

## 3 Results

The scores obtained in the diagnostic assessment showed no difference between the groups (Table 1; *p* > 0.05). In test 1, a significantly higher score was obtained for the active methodology group, compared to the traditional methodology group (Table 1; *p* < 0.05), while no difference between the groups was observed in test 2 (Table 1; *p* > 0.05). There was an increase from diagnostic to test 1 and to test 2 scores, in the traditional methodology (Table 1; *p* < 0.05). In the active methodology group, the mean score obtained in test 1 was higher compared to diagnostic assessment (Table 1; *p* < 0.05), without difference compared to test 2 (Table 1; *p* > 0.05).

**TABLE 1:** Cardiac physiology assessment scores obtained by the traditional teaching and active methodology groups.

Learning assessments	Traditional methodology^a^ (n = 51)	Active methodology^a^ (n = 52)
Diagnostic assessment	6.52 ± 2.60 A	6.36 ± 1.86 A
Test 1 (after only one of the teaching methods)	6.96 ± 2.00 B	8.89 ± 0.99 C
Test 2 (after the two teaching methods)	9.02 ± 2.00 C	8.94 ± 2.06 C

^a^ Mean ± standard deviation. Different letters indicate significantly different values (two-factor analysis of variance + Tukey’s test, *p* < 0.05).

**FIGURE 2:**
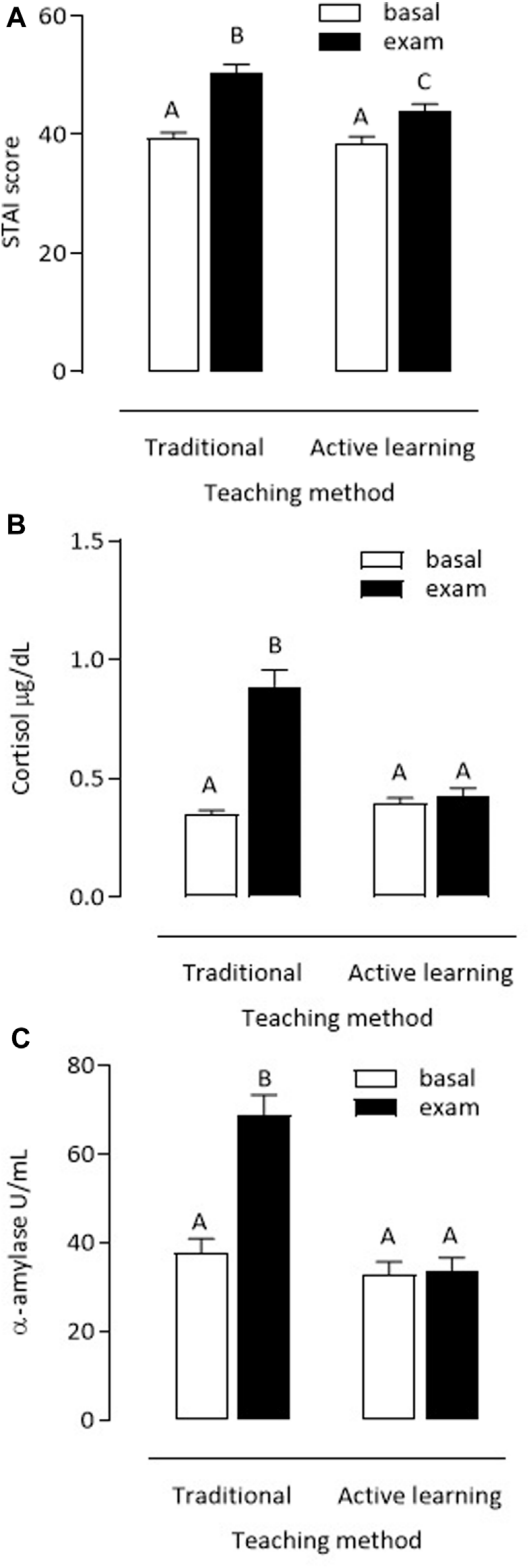
Student anxiety levels assessed using the STAI questionnaire **(A)** and saliva concentrations of the stress markers cortisol **(B)** and α-amylase **(C)**, before the tests applied after the cardiac physiology classes taught using the traditional (*n* = 51) or active (*n* = 52) methodologies. Different letters indicate significantly different values (two-factor analysis of variance + Tukey’s test, *p* < 0.05).

No significant differences between the groups were observed for the baseline anxiety score (Figure 2A), the concentration of cortisol (Figure 2B), or the concentration of α-amylase (Figure 2C) (*p* > 0.05).

The levels of anxiety increased before test 1, with a greater increase for the students who had received theoretical classes, compared to those who had studied the topic using the active methodology (*p* < 0.05; Figure 2A).

Before test 1, the concentrations of cortisol (Figure 2B) and α-amylase (Figure 2C) in saliva were higher for the theoretical class group, compared to both the baseline values and the values for the active methodology group (*p* < 0.05). For the active methodology group, these parameters showed no difference between baseline and prior to the test (*p* > 0.05; Figures 2B,C).

Although some of the students did not answer the questions concerning their perceptions, most of them considered that the activity with the educational game was useful for their learning, as well as that the learning assessments performed individually or as a group were useful for organizing their study (Table 2). The justifications provided by the students were grouped according to similarity. The three most frequent justifications, for each question, are shown in Table 2.

**TABLE 2:** Student perceptions of the teaching and evaluation strategies employed.

Question	Yes^a^	No^a^
Was the activity with the educational game useful for your learning?	93	3
Justifications		
*“It assisted me in understanding the content and remembering what I had already by studied*.*“(n* ^a^ *= 76)*		
*“We could learn as a group, with discussions within the group enabling us to comprehend what we had not previously by understood*.*“(n* ^a^ *= 33)*		
*“It helped me in identifying what I did not know and stimulated my by interest*.*“(n* ^a^ *= 10)*		
Did the individual and group tests change, in any way, your mode of studying and organizing your time?	80	12
Justifications		
*“They helped me to study continuously, not just before the test*.*” (n* ^a^ *= 55)*		
*“They helped in memorizing content by increasing my interest.” (n* ^a^ *= 9)*		
*“They helped in identifying my doubts before the test.” (n* ^a^ *= 8)*		

^a^ Number of students who gave this response. Some students did not answer some of the questions. Some of the students provided more than one justification, while some did not justify their responses.

## 4 Discussion

The findings demonstrated the positive effect of an active methodology on the learning of university students. The main contribution of this study is the evidence that the improvement of learning, using an active methodology, was accompanied by reduced levels of stress and anxiety before tests, when compared to traditional teaching with theoretical lectures.

Active methodologies stimulate the development of autonomy and engagement in students (Berbel, 2011; Randi and Carvalho, 2013; Borges and Alencar, 2014), with a positive effect on cognitive gain, defined as growth in knowledge, understanding, and cognitive skills linked to the desired learning results or course objectives (Rogaten et al., 2019). Studies have observed these effects in areas of knowledge including physics (Hoellwarth et al., 2005; Novilia et al., 2016), chemistry (Mathabathe and Potgieter, 2014), and physiology (Bhattacharya et al., 2017; Thomas et al., 2017). The increased learning observed here with the use of the educational game was consistent with previous work by our research group (Cardozo et al., 2016; Luchi et al., 2017; Luchi et al., 2019; Cardozo et al., 2020a) and other authors (Freeman et al., 2014; Gentry et al., 2019; Contreras-Arellano et al., 2020).

The effect on learning was evidenced in test 1, which was applied after the groups had received classes using one of the teaching methods. No difference was observed in the score obtained in the diagnostic assessment and in test 2, conducted after all the students had received classes using both methodologies. In the diagnostic assessment and test 2, a significant difference between the groups could possibly be related to the characteristics of the classes, which could explain the higher score obtained by the active methodology group in test 1. Therefore, these results reinforced that the active methodology resulted in better performance of the students, compared to the traditional method. Moreover, the absence of difference between test 2 and test 1 in active methodology group, and the score increase observed from test 1 to test 2 in the traditional methodology group, also support the interpretation that higher knowledge was provided by active methodology compared to traditional one.

Furthermore, this positive effect was accompanied by lower stress and anxiety before the tests. Given that the teaching-learning process is influenced by emotional factors (Relvas, 2012; Downing et al., 2020), this was an important finding, since it showed that use of the active teaching methodology can contribute to academic achievement, with greater satisfaction of the students regarding their chosen undergraduate course. Moreover, academic stress is a reality that many health students face, due to the demanding and complex nature of these courses. As they seek to assimilate a vast amount of information, develop practical skills, and face the pressure of rigorous assessments, these students often face heightened levels of stress. Unfortunately, this situation can have a profound impact on the learning process and the ability to retain information (Römer et al., 2011; Crego et al., 2016) because the release of stress hormones, such as cortisol, directly affect brain areas involved in learning. Also stress hormones indirectly alter the circuits used in learning through intermediate brain regions, such as the hippocampus, amygdala, and bed nucleus of the stria terminalis. These intermediate regions of the brain are not essential for the stress response or for learning but link the consequences of a stressful experience with circuits used to learn associations. Consequently, there is impairment in the formation of solid memories. In addition, academic stress can lead to a decrease in concentration and focus, crucial aspects for effective learning (Bangasser and Shors, 2010; Rossi et al., 2021). In this regard, the reduction of anxiety and stress has a significant impact on the learning process, memory, and performance of students. When anxiety levels are reduced, students can focus more effectively on academic tasks and learn more efficiently. Therefore, a less stressful learning environments may promote a positive attitude towards studying, allowing students to face challenges with more confidence (Kulasegaram and Rangachari, 2018; Carvalho et al., 2019; Dehaene, 2022). This scenario is possible when we use active teaching strategies combined with formative assessments (Cardozo et al., 2020b), as in the present study.

Different factors associated with active learning could explain the observed improvement in learning, together with the lower anxiety and stress when faced with assessments. One of these is the ability to accompany learning by means of formative assessments. This is different to the traditional method, where the teacher transmits the content, the student studies at home, and the acquired learning is assessed in a test. In the case of the active methodology used here, the formative formal (individual and group tests) and informal assessments (involving discussion with colleagues during the resolution of the educational game) enabled the students to perceive what they had understood and to identify their uncertainties (Hoffmann, 2014; Hoffmann, 2019). Formative assessments are fundamental in active learning methodologies, because they enable assessment of the teaching-learning process while it occurs (Kibble, 2007; Marcondes et al., 2021). In contrast, when only final summative assessment is applied, no activities or classes are subsequently performed to address issues that have been poorly understood. Hence, part of the knowledge that should have been acquired is not learned, which, during the undergraduate course, could compromise the confidence of the students in terms of their knowledge and skills.

Another factor inherent to the active learning methodology used here was the collaboration with colleagues in class activities aiming at the development of communication, argumentation, and teamwork skills (Redondo-Rodríguez et al., 2022). At the start of the semester, the teacher organized the groups in accordance with the norms of team-based learning (Bollela et al., 2014). Thus, the groups presented diversity in terms of gender and performance in the basic discipline delivered in the previous semester, including students who presented different degrees of difficulty, and avoiding the formation of groups based on friendships, for example,. This was explained to the students, pointing out that the objective was to promote development of the teamwork skills that would be required in their future careers, which should be independent of personal affinities. In collaborative learning, the student can perceive that other colleagues may also have doubts, so there is the possibility of being helped by colleagues at one moment, and helping them at another time (Caveião et al., 2018; Fragelli, 2019).

In addition, considering that the formation and reorganization of synaptic connections depends on the nature of stimuli, environmental conditions (Chidambaram et al., 2019), and emotional factors, it is essential that the teaching-learning process should occur in situations where the students feel at ease to express their doubts. A safe environment should be established for learning, making errors, and learning from the errors (Carvalho et al., 2019; Dehaene, 2022). These features can lead to greater confidence of students at the time of tests, explaining the observed reductions in anxiety and stress. The active learning strategy provided such a favorable environmental condition, as shown by the justifications of the students in their responses to the perception questionnaire. This condition would be hard to achieve using traditional theoretical lessons, where the student hears, sees, and receives information, without sufficient time to process it and identify doubts (Kulasegaram and Rangachari, 2018).

Another point that should be highlighted is that the group activities were planned and supervised by the teachers and monitors in such a way that all the members of each group had genuine participation, with their attention being focused on resolving the problems presented. Interventions and questions were made when it was observed that a student was inattentive, such as when a mobile phone was being used for a purpose unrelated to the learning activity. In this way, participation in the team was encouraged and it was sought to develop a sense of belonging, which can greatly contribute to academic success during university education. The approach adopted also aimed to minimize one of the problems often observed in the classroom, where young people have difficulty in focusing their attention and remaining interested and attentive during a class or activity (Rocha and Lemos, 2014). This is related to the fact that they consider themselves able to multitask, performing several activities at the same time. However, this is only possible when the simultaneous activities are automated and do not recruit cortical areas of the brain, examples being driving (which requires attention and is automated) and listening to music (which does not require focused attention). In contrast, paying attention in class, while at the same time answering a mobile phone message, requires the concurrent recruitment of cortical areas (Bear, 2017; Dehaene, 2022).

Beside these factors, the methodology included the application of a test prior to the activity with the educational game. The aim of this evaluation strategy was to stimulate individual study and preparation for the group activity in the classroom, with the students perceiving the need to take responsibility for their own learning (Cosenza and Guerra, 2011; Polydoro et al., 2015; Fior and Martins, 2020) and for the learning of their group. In addition, it contributed to better organization of the study routine, avoiding study shortly before the test, which frequently occurs when the traditional teaching method is used.

Contradictory to our positive results, several references in the literature found no significant difference in the performance of students submitted to active teaching methodologies when compared to traditional methods (Smallhorn, 2017; Knutstad et al., 2021; Sourg et al., 2023). Although the majority suggest an increase in student engagement and a positive attitude towards the learning method, no measurable increases in student learning outcomes were found. These studies do not mention by what means the application of active/innovative methodologies was conducted, the degree of collaboration between colleagues; or whether the activities were supervised by teachers, important points, as mentioned above. In addition, formative assessments were not applied, which proved to be a differential in the teaching-learning process in this work. The use of strategies that take the student out of the passive situation of receiving information, to situations that promote their active participation during the teaching-learning process does not necessarily result in better learning and training of undergraduates to apply the knowledge acquired in situation future (Cardozo et al., 2020a; 2020b).

At the start of each activity, the teacher explained the reason for the activity and why it was planned in a certain way. Questions were asked to stimulate reflection by the students, such as “Are you doing your part to learn?” or “Why did you not do the pre-class activity?”. The aim was to encourage metacognition in the students, so that they would think about their learning and their attitudes to developing it and enhance co-responsibility of teachers and students in the teaching-learning process (Dehaene, 2022).

It should also be noted that the pedagogical strategies and resources employed influence the outcomes of teaching and learning. The possibility of using a mixture of various activities and strategies broadens the spectrum of understanding, assimilation, and appropriation of diverse knowledge. This was evident in the present study, where it could be seen that the adoption of different teaching and assessment activities contributed to the students having a central role in the construction of their knowledge.

It could be concluded that the results obtained in the present study demonstrated the benefits of using an active methodology combined with formative assessments in the teaching-learning process. Furthermore, this approach also led to lower levels of anxiety and stress prior to learning assessments, when compared to the traditional teaching method.

## Data Availability

The raw data supporting the conclusion of this article will be made available by the authors, without undue reservation.
